# Workers’ perceptions of climate change related extreme heat exposure in South Australia: a cross-sectional survey

**DOI:** 10.1186/s12889-016-3241-4

**Published:** 2016-07-11

**Authors:** Jianjun Xiang, Alana Hansen, Dino Pisaniello, Peng Bi

**Affiliations:** School of Public Health, The University of Adelaide, Adelaide, 5005 SA Australia; Department of Emergency Response and Preparedness, Fujian Provincial Center for Disease Control and Prevention, Fuzhou, 350001 China

**Keywords:** Perceptions, Heat stress, Workplace heat exposure, Climate change, Work-related injuries

## Abstract

**Background:**

Occupational exposure to extreme heat without sufficient protection may not only increase the risk of heat-related illnesses and injuries but also compromise economic productivity. With predictions of more frequent and intense bouts of hot weather, workplace heat exposure is presenting a growing challenge to workers’ health and safety. This study aims to investigate workers’ perceptions and behavioural responses towards extreme heat exposure in a warming climate.

**Methods:**

A cross-sectional questionnaire survey was conducted in 2012 in South Australia among selected outdoor industries. Workers’ heat risk perceptions were measured in the following five aspects: concerns about heat exposure, attitudes towards more training, policy and guideline support, the adjustment of work habits, and degree of satisfaction of current preventive measures. Bivariate and multivariate logistic regression analyses were used to identify factors significantly associated with workers’ heat perceptions.

**Results:**

A total of 749 respondents participated in this survey, with a response rate of 50.9 %. A little more than half (51.2 %) of respondents were moderately or very much concerned about workplace heat exposure. Factors associated with workers’ heat concerns included age, undertaking very physically demanding work, and the use of personal protective equipment, heat illness history, and injury experience during hot weather. Less than half (43.4 %) of the respondents had received heat-related training. Workers aged 25–54 years and those with previous heat-related illness/injury history showed more supportive attitudes towards heat-related training. The provision of cool drinking water was the most common heat prevention measure. A little more than half (51.4 %) of respondents were satisfied with the current heat prevention measures. About two-thirds (63.8 %) of respondents agreed that there should be more heat-related regulations and guidelines for working during very hot weather. More than two-thirds (68.8 %) of the respondents were willing to adjust their current work habits to adapt to the likely increasing extreme heat, especially those with previous heat illness experience.

**Conclusions:**

The findings suggest a need to strengthen workers’ heat risk awareness and refine current heat prevention strategies in a warming climate. Further heat educational programmes and training should focus on those undertaking physically demanding work outdoors, in particular young workers and those over 55 years with low education levels.

**Electronic supplementary material:**

The online version of this article (doi:10.1186/s12889-016-3241-4) contains supplementary material, which is available to authorized users.

## Background

Occupational exposure to extreme heat without sufficient protection may not only increase the risk of heat-related illnesses and injuries [1–5], but also compromise economic productivity by reducing work efficiency [6] and the time that work can safely be undertaken outdoors during the hottest part of the day [7]. Workplace heat gain can be a combination of heat from the external thermal environment and internal heat generation by metabolism associated with physical activity [8]. External heat exposure sources in the workplace include weather-related and process-generated heat. With predictions of more frequent and intense bouts of hot weather [9], workplace heat exposure is presenting a growing challenge to workers’ health and safety [10–12], especially for outdoor workers and those undertaking physically demanding work in hot environments [13].

Evidence shows that Australia would be at moderate to high risk of occupational heat strain for outdoor workers if temperatures increase by 3 °C [14]. Dangerous days (days when there is a 2.5 °C increase in body temperature in less than 2 hours) for Australian outdoor workers may increase to 15–27 days per year by 2070 compared with 1 day per year at present [15]. Several heat-related deaths have recently been reported in Australian workplaces [16–18], raising increased concerns over workplace heat exposure. The potential heat-related impact of climate change on workplaces may be even worse in South Australia (SA) than the Australian national average level, as the average maximum temperature has increased at a faster rate than the national average since 1950 [19]. Adelaide, the capital of SA, has a Mediterranean climate, characterised with very limited rainfall during hot dry summers and maximum temperatures reaching as high as 46.1 °C. According to weather projections for Adelaide, the average number of days with temperatures over 35 °C will triple by 2070 [19]. Some studies in Adelaide have found that heat stress levels were high in some occupations (e.g., miners, shearers, and road maintenance workers) [13], and work-related injury claims were significantly associated with ambient temperatures and heatwaves [2, 3].

Heat-related illnesses and injuries are largely preventable. Current heat prevention strategies include engineering controls, administrative control, personal protection, education and training, and regulations [8]. Effective heat stress management needs comprehensive efforts, cooperation, and support from a wide variety of stakeholders such as employees, employers, and occupational health professionals. An investigation of how people perceive the risk of workplace heat exposure may be helpful to identify potential heat prevention and adaptation barriers, to make the allocation of heat prevention resources more focused, and to refine current heat policies to make them more practicable and operational.

To raise awareness of the harm caused by weather-related extreme heat exposure, the US Occupational Safety and Health Administration lunched the national-wide Heat Illness Prevention Campaign in 2011 [20]. Prior to the possible development of heat stress management, there is a need to investigate the baseline information regarding how people perceive the risk of heat stress. To the authors’ knowledge, only two qualitative studies have preliminarily explored Australian workers’ heat risk perceptions [11, 21], and suggested that there was a misalignment of perceived occupational health risk, given Australia’s existing thermal environments and predicted increasingly hot summers [9]. The present quantitative study using a questionnaire survey is the first in Australia with aims to investigate workers’ perceptions and behavioural responses regarding extreme heat exposure, and identify factors affecting individuals’ heat perceptions.

## Methods

A cross-sectional questionnaire survey was conducted between 15 August and 6 November 2012 in Adelaide, South Australia, amongst selected workers and trades apprentices at high risk of workplace heat exposure. The average daily maximum temperature was 20.6 °C in Adelaide during the study period, ranging from 12.4 to 35.0 °C.

### Questionnaire design

The questionnaire was developed after a comprehensive review of the literature on heat exposure and occupational health [13]. The draft questionnaire was reviewed by relevant experts and piloted among 10 outdoor workers in Adelaide. Relevant revisions were made to ensure all questions were clear and understandable.

The final questionnaire (Additional file 1) consisted of 5 demographic variables and 20 questions involving aspects of the working environments, previous history of heat-related illness and injury, heat prevention management, and perceptions of workplace extreme heat exposure. Some questions had both closed and open-ended responses. Some multiple choice questions investigated individual work habits, access to heat stress prevention information, and heat prevention measures. Likert-scale questions were used to measure heat-related perceptions and attitudes.

### Participant recruitment

The inclusion criteria for participants were those working outdoors, or indoors without air conditioning, in the following four industries: “agriculture, fishing and forestry”, “construction”, “electricity, gas and water” (the latter refers to workers engaged in the provision of public utilities), and “mining”, according to the findings of previous studies [2, 3] and relevant literature [13].

With the support of SafeWork South Australia (SWSA), the local occupational health and safety (OH&S) regulator, a total of 164 employers who reported relatively more injury claims during the period of 2001–2010 in SA were invited to participate in the study. These employers provided assistance in the distribution of survey questionnaires, envelopes, and information sheets (Additional file 2) to their workers who met the inclusion criteria.

Young workers appear to be relatively at higher risk of heat-related injuries in the workplace [22], however they may be underrepresented due to the aging workforce. Moreover, young workers were usually less inclined to participate in surveys due to various reasons. Therefore, apprentices at local TAFE (Technical and Further Education) colleges were also recruited to represent young workers, a large proportion of whom undertook their studies part time while working as apprentices. In total, 840 TAFE trainees and apprentices enrolled in the courses likely to lead to outdoor work in the “agriculture, forestry and fishing”, “construction”, “electricity, gas and water”, “manufacturing”, and “mining” industries were invited to participate in the survey. After initial contact and meeting with the TAFE College management team and relevant lecturers, questionnaires were distributed by the course lecturers.

Participants filled out the questionnaires independently in their own time. Completed questionnaires were returned by participants using supplied reply-paid envelopes. All participants were therefore free of any potential pressure from their employers and supervisors during the completion of the questionnaire. Oral consent was obtained from each participant. The study was approved by the Human Research Ethics Committee at the University of Adelaide (H-200-2011).

#### Data analyses

Data entry and validation were performed using ‘Microsoft Excel 2007’, and imported into Stata statistical software (version 12.0, College Station, Texas, USA) for data manipulation and analyses. The “SVY” commands of Stata were used to calculate odds ratios (OR) and 95 % confidence intervals for the prevalence estimates [23].

Five indicators were used to represent workers’ perceptions of workplace heat exposure from different aspects. They were (1) workers’ concerns about heat exposure, (2) attitudes towards more training, (3) policy and guideline support, (4) the adjustment of work habits, and (5) degree of satisfaction of current preventive measures. To identify the factors significantly associated with perceptions of workplace heat exposure, bivariate and multivariate logistic regression analyses were conducted using a stepwise backwards model. All variables with statistical significance of *p* < 0.05 were included in the final model.

## Results

### Demographic characteristics

A total of 1,471 questionnaires were distributed and 749 were returned, with a response rate of 50.9 %. The 749 respondents consisted of 511 (68.2 %) TAFE apprentices and 238 (31.8 %) established workers. Among the 511 TAFE apprentices, 91.4 % were part-time, with 8.6 % full-time TAFE students. As shown in Table 1, the majority (96.0 %) of respondents were male, and young people (≤24 years) accounted for more than half (53.5 %) of all respondents. In terms of highest educational attainment, more than half (51.3 %) of the respondents had completed high school, 42.6 % had a trade certificate, and 6.1 % had a university degree. Almost three-quarters (73.2 %) of respondents were employed as tradespersons and related workers. Labourers accounted for 5.7 % of respondents. More than half (51.0 %) of respondents mainly undertook work outdoors. The construction industry had the highest percentage (37.1 %) of respondents, followed by manufacturing (27.5 %), mining (14.6 %), ‘electricity, gas and water’ (13.1 %), and ‘agriculture, forestry & fishing’ (2.0 %). Approximately three-quarters (75.9 %) of respondents considered that their jobs were moderately or highly physically demanding, while 38.1 % reported that they worked close to heat sources. Two-thirds (67.8 %) of respondents were required to wear personal protective equipment (PPE), which may include overalls, gloves, helmets, goggles, respirators, face masks, and high visibility clothing etc.

**Table 1:** Perceptions of workplace heat exposure: prevalence estimates and 95 % CI by different subgroups

Independent variable	*n*	%	Concern for extreme heat % (95 % CI)	Positive attitude for more training % (95 % CI)	Positive attitude for more regulation % (95 % CI)	Positive attitude for adjusting work habits % (95 % CI)	Satisfaction degree for preventive measures % (95 % CI)
Total	749	100	51.2 (50.0–57.4)	56.3 (50.1–61.9)	63.8 (57.3–69.9)	68.8 (63.4–73.7)	51.4 (42.6–60.0)
Gender							
Male	719	96.0	51.1 (44.7–57.5)	56.5 (51.1–61.7)	64.1 (57.5–70.2)	69.2 (63.9–74.0)	50.9 (42.3–59.5)
Female	30	4.0	53.3 (34.1–71.6)	53.3 (28.9–76.3)	56.7 (37.7–78.9)	60.0 (40.5–76.8)	62.1 (39.8–80.2)
Age group							
≤24	401	53.5	44.0 (38.5–49.6)	50.0 (43.1–56.9)	66.9 (58.7–74.3)	66.4 (59.2–73.0)	42.0 (35.6–48.7)
25–34	101	13.5	58.5 (48.9–67.5)	63.5 (56.4–70.1)	74.0 (63.8–82.0)	76.3 (64.0–85.5)	44.1 (31.7–57.3)
35–54	161	21.5	63.5 (48.3–76.4)	66.7 (53.2–77.9)	59.0 (48.2–69.0)	73.7 (63.2–82.1)	68.8 (58.1–77.9)
≧55	86	11.5	59.8 (44.3–73.5)	64.6 (46.5–79.4)	48.8 (37.2–60.5)	65.9 (51.3–78.0)	70.9 (55.7–82.5)
Education level							
High school	384	51.3	46.8 (40.7–53.0)	53.4 (46.6–60.1)	66.2 (58.4–73.2)	67.6 (60.5–73.9)	46.4 (37.1–55.9)
Trade certificate	319	42.6	56.1 (48.5–63.3)	58.7 (52.6–64.5)	64.0 (56.7–70.7)	69.8 (63.5–75.4)	51.8 (42.0–61.5)
University degree	46	6.1	53.3 (43.4–63.0)	62.2 (49.3–73.6)	42.2 (24.1–62.7)	73.3 (52.8–87.1)	86.7 (71.9–94.3)
Occupation							
Tradespersons and related workers	548	73.2	50.5 (44.5–56.4)	55.1 (49.5–60.6)	66.7 (59.9–72.8)	70.0 (65.3–74.4)	46.6 (38.5–55.0)
Clerical and administrative workers	30	4.0	40.0 (19.8–64.2)	70.0 (28.1–93.3)	46.7 (21.6–73.6)	66.7 (36.6–87.4)	86.2 (64.8–95.5)
Machinery operators and drivers	55	7.3	65.5 (32.1–88.4)	58.2 (27.8–83.4)	61.8 (36.9–81.7)	54.6 (42.9–65.7)	63.6 (42.7–80.4)
Labourers and related workers	43	5.7	51.2 (32.5–69.5)	55.8 (47.7–63.7)	62.8 (38.3–82.1)	69.8 (54.4–81.7)	45.2 (30.0–61.4)
Full-time TAFE students	44	5.9	40.9 (21.0–79.0)	54.6 (39.6–68.7)	59.1 (48.3–69.1)	67.4 (53.4–78.9)	56.1 (35.6–74.7)
Professionals	16	2.1	81.3 (62.0–92.0)	68.8 (30.0–91.9)	37.5 (15.7–65.9)	81.3 (53.1–94.3)	87.5 (51.5–97.9)
Other	13	1.7	46.2 (17.2–77.9)	61.5 (24.9–88.5)	46.2 (16.7–78.6)	69.2 (37.7–89.3)	76.9 (34.1–95.6)
Industry							
Agriculture, forestry & fishing	15	2.0	50.0 (24.4–75.6)	62.5 (41.3–79.8)	66.7 (49.4–80.4)	58.3 (37.2–76.8)	69.6 (51.6–83.0)
Mining	109	14.6	57.0 (45.5–67.8)	51.3 (35.2–67.2)	59.1 (38.4–77.0)	67.3 (44.9–83.8)	59.5 (32.6–81.7)
Manufacturing	206	27.5	44.2 (37.9–50.8)	49.5 (32.5–66.7)	67.5 (45.7–83.6)	66.7 (48.7–80.8)	36.4 (25.8–48.6)
Construction	278	37.1	52.4 (38.8–65.6)	58.8 (51.6–65.6)	67.0 (59.5–73.8)	69.3 (63.9–74.3)	50.2 (43.2–57.2)
Electricity, gas & water	98	13.1	57.6 (33.3–78.7)	67.9 (60.7–74.4)	52.8 (43.4–62.1)	75.5 (64.4–84.0)	71.7 (43.8–89.2)
Other	43	5.7	38.5 (9.4–78.9)	53.9 (28.8–77.1)	61.5 (23.8–89.1)	69.2 (35.6–90.1)	46.2 (12.8–83.3)
Workplace environment							
Completely indoors	59	7.9	45.7 (37.0–54.8)	74.6 (57.5–86.4)	71.2 (53.9–83.9)	69.0 (52.1–82.0)	37.3 (21.4–56.5)
Mainly indoors	282	37.7	44.4 (35.7–53.6)	52.5 (43.0–61.8)	58.5 (48.4–68.0)	68.6 (62.7–74.0)	59.5 (44.6–72.8)
Completely outdoors	82	10.9	71.6 (55.7–83.5)	63.4 (49.5–75.4)	70.7 (58.6–80.5)	63.8 (46.3–78.2)	44.3 (29.9–59.7)
Mainly outdoors	300	40.1	53.2 (48.6–58.3)	55.3 (50.0–60.6)	63.7 (57.1–69.7)	68.9 (61.6–75.4)	49.0 (41.5–56.5)
Physically demanding							
Not at all	49	6.5	40.4 (29.3–52.7)	63.8 (38.8–83.1)	51.1 (32.4–69.4)	73.9 (61.8–83.2)	82.6 (69.0–91.0)
A little	132	17.6	44.5 (35.9–53.5)	53.1 (39.1–66.6)	47.7 (35.5–60.2)	59.8 (47.3–71.2)	70.6 (53.5–83.4)
Moderately	280	37.4	48.0 (40.3–55.8)	56.1 (50.3–61.8)	64.0 (56.6–70.8)	72.0 (65.4–77.7)	50.9 (44.8–57.0)
Very much	288	38.5	59.9 (50.7–68.4)	56.6 (50.0–63.1)	72.7 (66.3–78.4)	69.5 (63.9–74.6)	37.8 (30.0–46.5)
Work close to heat sources							
Yes	285	38.1	52.4 (46.7–58.0)	56.8 (49.9–63.4)	67.0 (59.1–74.0)	69.8 (64.9–74.2)	44.8 (35.6–54.4)
No	464	61.9	49.3 (39.8–58.8)	56.5 (46.5–66.0)	59.3 (50.8–67.3)	68.4 (60.1–75.6)	61.7 (50.5–71.7)
Use of personal protective equipment							
Yes	508	67.8	55.8 (49.7–61.8)	58.0 (52.4–63.3)	64.2 (58.0–70.0)	69.9 (64.4–74.8)	50.1 (40.5–59.7)
No	241	32.2	41.4 (34.0–49.4)	52.9 (45.0–60.7)	62.9 (49.7–74.5)	66.5 (58.1–74.0)	54.1 (40.7–66.9)
Heat illness experience							
Yes	279	37.2	62.4 (52.2–71.5)	65.2 (56.8–72.8)	70.3 (62.9–76.7)	73.9 (67.8–79.2)	38.5 (30.6–47.0)
No	470	62.8	44.5 (39.5–49.6)	51.4 (44.2–58.5)	60.4 (52.9–67.4)	66.1 (60.1–71.6)	59.0 (48.2–69.1)
Heat-related injury experience							
Yes	194	25.9	67.0 (57.0–75.7)	65.0 (57.0–72.1)	72.2 (60.6–81.4)	71.2 (62.7–78.4)	29.5 (23.5–36.2)
No	555	74.1	45.6 (39.9–51.5)	53.3 (47.5–59.1)	60.9 (54.0–67.4)	68.0 (62.5–73.0)	59.0 (48.8–68.5)

### Heat-related illnesses and injury experience during very hot weather

Overall, 279 (37.2 %) of respondents had experienced heat illnesses during hot days. The most common type of heat illnesses reported were heat exhaustion (60.6 %), followed by heat rashes (43.0 %), heat stroke (26.2 %), and heat cramps (18.3 %) (Data not shown, Question 12, Additional file 1). About one-quarter (25.9 %) of respondents experienced heat-related injuries during very hot weather. More than half (54.1 %) of the injuries were caused by burns, 44.3 % by falls, trips and slips, 27.8 % by hitting objects, and 10.3 % by being hit by moving objects (Question 15, Additional file 1).

When asked if they had witnessed an injury to another person during hot weather (Question 16, Additional file 1), 25.2 % of participants responded that they had. The most common type of injuries witnessed during very hot weather was falls, trips and slips (55.0 %), followed by burns (42.3 %), hitting objects (22.8 %), and being hit by moving objects (17.5 %).

### Risk perceptions of workplace heat exposure

Overall, 51.2 % of respondents were moderately or very much concerned about the risk of heat illness at work during very hot weather (Table 1). Results of stepwise logistic regression analyses indicated that workplace heat concern levels increased with the increasing age. As shown in Table 2, workers in the three age groups (25–34, 35–54, and ≥55 years) were respectively 1.82 (95 % CI 1.18-2.80), 2.73 (95%CI 1.45-5.12), and 2.77 (95 % CI 1.73-4.43) times more concerned about heat stress than those aged ≤24 years. Other factors significantly associated with workers’ heat stress concerns include undertaking heavily physically demanding work (OR = 2.77, 95 % CI 1.73-4.43), wearing PPE (OR = 1.47, 95 % CI 1.13-1.91), having a history of heat illness (OR = 1.57, 95 % CI 1.01-2.45) and having experienced an injury during hot weather (OR = 2.05, 95 % CI 1.47-2.86).

**Table 2:**
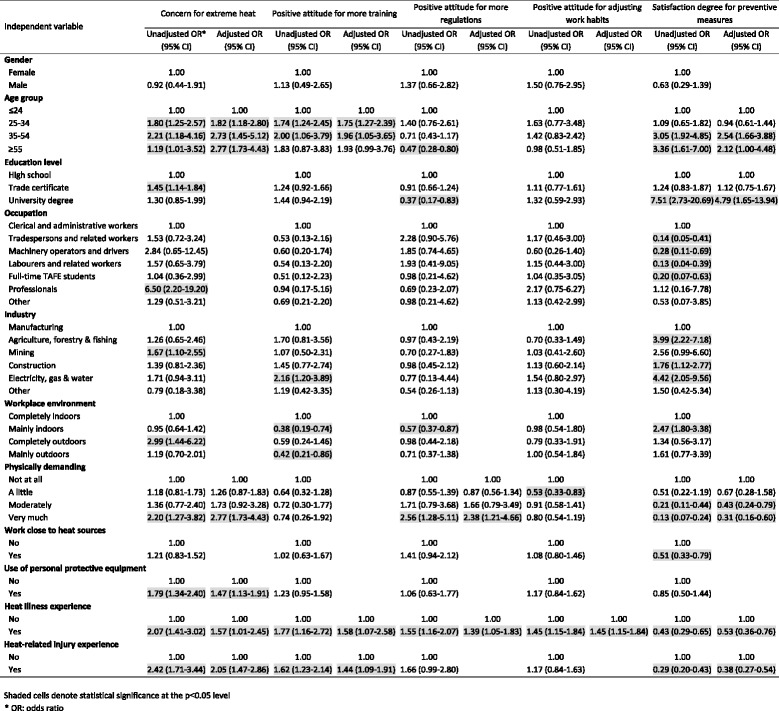
Factors associated with attitude and perception for workplace heat exposure: bivariate analysis (unadjusted) and multiple stepwise logistic regressions (adjusted)

About 56.3 % of respondents replied that there was a need for more heat-related training for workers to reduce the risk of heat stress (Table 1). As shown in Table 2, workers aged 25–54 years showed more supportive attitudes towards heat-related trainings than younger (≤24 years) and older workers (≥55 years). In addition, workers with previous history of heat illness (OR = 1.58, 95 % 1.07-2.58) and injury during hot weather (OR = 1.44, 95 % 1.09-1.91) were more interested in heat-related trainings.

Results showed that 63.8 % of respondents agreed that there should be more heat-related regulations and guidelines for working during very hot weather (Table 1). Regarding the reasons why the remaining 36.2 % held the opposite view, answers from a subsequent question (Question 20, Additional file 1) suggested that over half (52.3 %) thought “there are enough heat regulations”, while 21.5 % considered workplace heat exposure to not be a serious problem. Multiple logistic regression analyses suggested that very physically demanding work (OR = 2.38, 95 % CI 1.21-4.66) and heat illness experience (OR = 1.39, 95 % CI 1.05-1.83) were the two factors associated with workers’ attitudes toward more heat-related policy support (Table 2).

More than two-thirds (68.8 %) of respondents answered they were willing to adjust their current work habits to adapt to the impact of extreme heat (Table 1). As to the reasons the remaining 31.2 % did not consider the adjustment of work habits during hot weather (Question 24, Additional file 1), one-third (33.3 %) thought “Enough has been done already”, followed by “I don’t think I am at risk” (30.3 %), and “I don’t think it is a serious problem” (24.4 %). Multiple logistic regression analyses indicated that workers with previous heat illness experience (OR = 1.45, 95 % CI 1.15-1.84) showed stronger willingness to adjust their heat-related work habits.

More than half (51.4 %) of respondents were satisfied with the heat prevention measures currently adopted in South Australian workplaces (Table 1). Factors significantly associated with workers’ satisfaction in terms of current heat prevention measures include: age (35–54 and ≥55 years), education level (university degree), performing physically demanding work, previous heat illness experience and injury experience during hot weather (Table 2).

### Personal behaviours during hot days

The majority (64.4 %) of respondents drank water regularly during work, while 16.4 % responded that they only drank when feeling thirsty, and 15.2 % answered that they drank plenty of fluids before starting work. About one-fifth of participants’ claimed all of these drinking habits in the workplace were applicable (Question 10, Additional file 1).

**Fig. 1:**
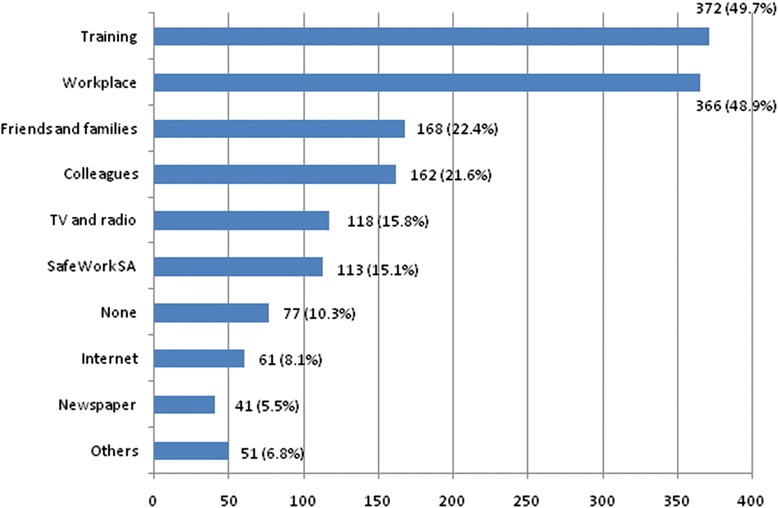
Main sources of information about heat prevention

In terms of the main sources of information about heat prevention, as shown in Fig. 1, heat-related training (49.7 %) and learning at the workplace (48.9 %) were the most common way for respondents to obtain such information, followed by information from friends and families (22.4 %), colleagues (21.6 %), TV and radio (15.8 %), SafeWork SA (15.1 %), the internet (8.1 %), and newspapers (5.5 %). Some 10.3 % of respondents stated that they could not access any information about heat stress prevention.

Respondents were asked if they worked at their own pace during very hot weather and the majority (70.4 %) answered in the affirmative. As to the reasons why the remaining 29.6 % did not work at their own pace (Question 23, Additional file 1), more than two-thirds (68.0 %) attributed this to the pressure from work demands, followed by the pressure from supervisors (46.1 %), and peer pressure (24.3 %). In addition, 11.2 % replied that there was no need to slow down their work rate during hot weather because “Enough has been done to cool the workplace”.

### Current heat prevention measures

**Fig. 2:**
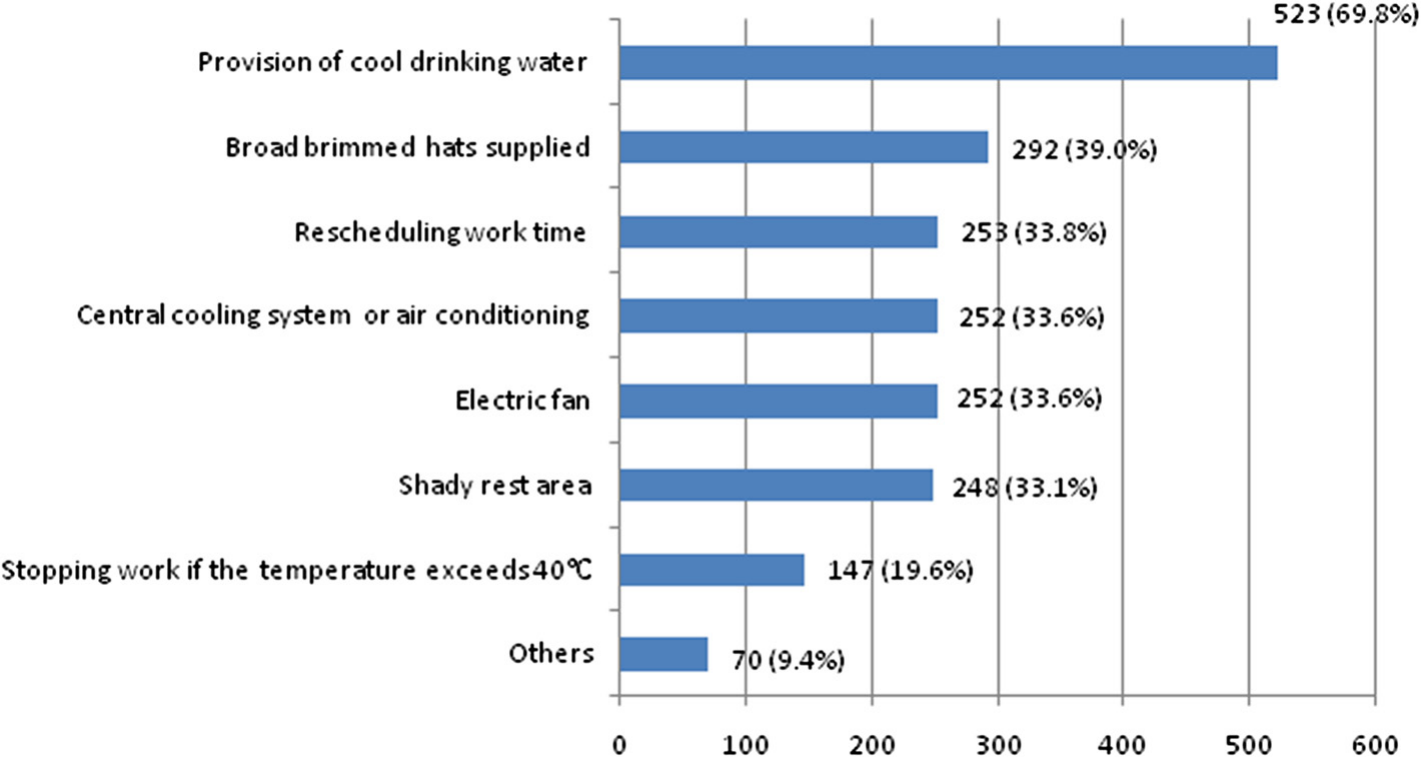
Heat prevention measures currently adopted in the workplace

As shown in Fig. 2, the provision of cool drinking water (69.8 %) was the most common prevention measure adopted in the workplace against heat exposure in South Australia, followed by wearing broad brimmed hats (39.0 %), rescheduling work time (33.8 %), central cooling and air conditioning (33.6 %), electric fans (33.6 %), and shady rest area (33.1 %). Only 19.6 % answered “stopping work” as a prevention measure when the temperature was extremely hot (e.g., >40 °C).

When asked if there were guidelines for heat stress prevention during extremely hot weather, 50.9 % of respondents answered “Yes”. Whilst 43.4 % had attended a heat-related training course, 60.0 % participants had received instructions on first aid procedures for serious heat illnesses.

## Discussion

It is important to understand workers’ perceptions on workplace extreme heat exposure in a warming climate, as this information may provide evidence for updating heat prevention strategies to reduce the impact of climate change on workers’ health and safety. However, most of the currently available information is from qualitative studies in middle-low income countries [24–26]. Limited evidence suggests that workers’ perceptions regarding likely increasing extreme heat exposure due to climate change in high income countries are not optimistic [11]. This survey is a modest step to investigate workers’ heat risk perceptions in a developed country, using a quantitative approach.

### Workplace heat exposure concern in Australia

This study revealed that about half of the respondents were moderately to very much concerned about their occupational health and safety when working during very hot weather. The percentage (50 %) concerned in those participants employed in the “agriculture, forestry and fishing” industry in this study was about 6 times higher than that (8 %) in a study of Californian hired farmworkers in the USA [27]. The relatively higher heat awareness among the Australian workplace may reflect cultural and demographic differences in study populations.

Our results suggest that young workers were less concerned about heat exposure than older workers. Moreover, middle aged workers supported having more heat-related training compared to young and older workers. There are many concerns about Australian young workers’ attitudes towards occupational health and safety, of which heat stress is only one. According to the national “Motivations, Attitudes, Perceptions and Skills” survey, up to 28 % of Australian young workers reported resenting dealing with workplace health and safety requirements, and 42 % forgot about safety during working practice [28]. Evidence has shown that young workers are at high risk of heat-related illnesses and injuries when the temperature is below a certain threshold (e.g., 37.9 °C) [2, 4, 5]. By contrast, older workers are more vulnerable to heat-related illnesses/injuries in certain outdoor industries during heatwaves [3]. Therefore, more educational programmes should be targeted to these two age groups. Further research is also needed to explore the paradoxical phenomena that young workers were less satisfied with heat prevention measures but expressed more negative attitudes towards heat prevention efforts, compared with older workers. Probably, it is because young workers were relatively more powerless than older workers in the workplace and held negative attitudes towards their management [21]. The compliance and implementation of heat prevention and adaptation policies would be undermined if the sentiments of unwillingness to cooperate existed among young workers [28].

Undertaking very physically demanding work and wearing PPE was also found to be associated with workers’ concern about heat exposure. Both of these are very important factors determining human body heat balance, and can be indirectly reflected in the standard workplace heat stress management procedure [8]. The results also suggest that workers who had a previous heat-related illness and/or injury were more concerned about heat exposure.

### Heat-related training

In this study, about 43 % of respondents indicated that they have received heat-related training. The relatively higher proportion of workers receiving heat prevention training in this study maybe partly because of the lessons learnt from the past. Previously, less OH&S training and education had been provided in Australian workplaces, and this was identified as one of the top three causes of occupational injuries and accidents in Australia [29]. Therefore, relevant work health and safety training has been incorporated into secondary, vocational and university level education since the mid-to late 1990s in Australia [29].

The results indicated that heat training was the workers’ major source of information about heat stress prevention in Australia, and this is the case for occupational health and safety in general [29]. Evidence has suggested that the majority of outdoor physical workers in the USA [27, 30], India [26] and South Africa [24] had a good knowledge of the symptoms and severe outcomes of excessive heat exposure [24, 27, 30]. Although workers’ average level of knowledge on heat stress wasn’t investigated in this study, 16 % of respondents said they only drank when they were thirsty. This indirectly reflects the necessity to reinforce messages about dehydration in the workplace. Relevant training and education should focus on young workers and those aged over 55 years, as these groups expressed less willingness to receive more heat training. Moreover, studies have shown they are at relatively higher risk of heat-related illness and injury [2–4]. More supportive attitudes towards heat-related training in the age group of 25–54 years may account for their greater concerns over heat exposure. The reasons why workers aged ≥55 years did not show stronger willingness to support more heat-related training whilst being more concerned about heat compared to young workers, may include that they may be more satisfied with current preventive measures in place or they may undertake more sedentary jobs with less heat exposure. In addition to training in the workplace, the role of mass media in popularizing heat stress prevention knowledge should be strengthened, as up to 10 % of respondents in this study claimed to have had no sources of heat prevention information.

### Individual behavioural response

The majority of respondents expressed their willingness to adjust work habits to adapt to possible increasing hot weather, and this may be useful for future heat intervention measures. The results from this study suggested that previous heat illness experience was the only factor associated with the adjustment of work habits, indicating a need for improving heat risk awareness. Moreover, a good level of heat stress knowledge and awareness does not necessarily translate into individual behavioural change [30]. The knowledge-behaviour gaps may provide opportunities for additional heat prevention and education strategies.

In other studies, self-pacing (adjusting work rate to avoid physiological heat strain) has been used to explain why workers were not heat stressed when working in hot environments [13, 31]. In the present study, up to 70 % of respondents expressed that they worked at their own pace during very hot weather. For others, pressure from work demand and supervisors was the major reason that workers did not slow down their work rate. Most recently, Lao et al. interviewed 32 male council workers in South Australia and found they had a high level of heat resilience through personal adaptive behaviours [21]. Nevertheless, profit-oriented production and performance targets have been shown to be a common reason overshadowing or marginalizing heat stress prevention [11, 21]. Employee-based behavioural change is not enough to reduce heat-related illness and injury, as employees may be powerless in an occupational health and safety management system [11, 21, 32]. Relevant heat prevention campaigns and legislations should target employers as heat stress not only impacts workers’ health and safety but also may compromise productivity [1, 6, 11, 12, 33]. However, to date few studies have investigated how employers in industry perceive the risk of heat exposure, although results of our recently published paper showed that the majority of occupational hygienists and specialists in Australia were concerned about workplace extreme heat exposure [34].

### Heat prevention measures

In this study about one-quarter of respondents claimed to have witnessed heat-related illnesses or injuries during extremely hot weather. This may indirectly reflect the high incidence of occupational heat illnesses despite only 306 heat-related compensation claims being identified in South Australia during the period of 2001–2010 with an incidence rate of 4.5 per 100 000 employees [35]. This figure may be underestimated due to underreporting and misclassification [13]. Fortunately, all heat-related illnesses, injuries and deaths are largely preventable. Heat prevention strategies mainly include regulations, administrative controls, and engineering modifications. Currently, there are systematic technical guidelines and manuals in place for heat stress monitoring, risk assessment, control and prevention, such as ISO (International Organization for Standardization) heat indices [36], ACGIH (American Conference of Governmental Industrial Hygienists) [37] and NIOSH (US National Institute for Occupational Safety and Health) heat standards [38]. Based on above heat standards, the Australian Institute of Occupational Hygienists (AIOH) has developed a heat stress management guideline for use in the Australian environment [8]. However, it should be realized that these guidelines and technical manuals do not have a legal force.

It has been proven that without proper enforcement heat regulations are likely to pose little restrictions to non-compliant employers [39, 40]. In 2010, California became the first State in the USA to enact a stringent heat-specific law to protect workers from heat exposure [41]. Two years later however, inspectors found that more than half of the employers they audited did not comply with the heat standard [42]. Heat prevention measures seem straightforward, common-sense, and simple (e.g., drinking water frequently, wearing light coloured and permeable clothes, taking breaks in the shade, and responding to early symptoms). However, a variety of factors at multiple levels in the workplace may constrain such implementation, such as production quotas, worries of being considered ‘soft’, and workers’ fears of losing their job [11, 21, 39]. Currently, there is no federal occupational standard specifically addressing heat illness and injury prevention in Australia [12, 35], which may make the implementation of heat prevention measures problematic.

Maintaining hydration is very important for heat prevention. In this study, approximately 30 % of respondents replied that cool drinking water was not available in the workplace. Moreover, about 16 % of respondents only drank when thirsty. Thirst cannot be relied upon as a guide for the need for water, as 1 % of the total body weight in water is already lost when an individual senses thirst [43]. According to the national Model Code of Practice (managing the work environment and facilities) [44], “an adequate supply of clean drinking water must be provided free of charge for workers at all times.” However, its implementation and effectiveness are questionable, as evidence has shown that a poor hydration status has been observed among workers employed in a range of industries in Australia [45, 46].

Our results showed that only 20 % of respondents selected “stopping work” as a heat prevention measure when the temperature was extremely hot. However, according to the heat stress management policy of the Construction, Forestry, Mining and Energy Union (CFMEU) South Australia Branch, “if temperature is over 37 °C all work ceases unless working in an air conditioned area” [47]. In this study, the majority (67 %) of participants were recruited from agriculture, forestry, mining, construction, and “electricity, gas and water” industries, and about half worked outdoors. Therefore, this raises concerns regarding the compliance of heat policies.

In this study, about 64 % of respondents thought there was a need for more heat-related regulations. Meanwhile, about half the respondents were not satisfied with current prevention measures, indicating the necessity and urgency of the development of heat policies, especially for young workers with low education levels and undertaking physically demanding work outdoors. Most recently, Jia and Rowlinson et al. [48] formulated a comprehensive socio-ergonomic framework for identifying heat risk factors in the construction industry, and suggested a set of localized, simplified, action-triggering and threshold-based guidelines for the development of current heat management system [49]. This may provide useful inspirations for developing more effective and practical heat stress management for the construction industry and other at-risk industries. Therefore, in addition to strengthening the implementation of current existing regulations during extremely hot days, more efforts are needed to develop local industry specific heat stress policies [49].

### Limitations

There are several limitations in this study. First, the vast majority (96 %) of respondents were males. Caution should be used when generalizing the results to female workers. Moreover, as the participation of the survey was completely voluntary, those with previous heat illness and injury experience may have been more likely to participate in the survey, which may generate potential selection bias and therefore may overestimate workers’ heat concerns. Second, the level of workers’ heat-related knowledge was not specifically measured in this study. However, published papers have consistently found that most workers in both developing and developed countries have a good knowledge of heat illnesses [24, 27, 30, 50]. Third, the relatively low response rate (50.9 %) in this study may generate potential non-respondent bias. Although there is not necessarily a relationship between response rates and bias, non-response bias may occur if respondents significantly differ from non-respondents [51]. Fourth, 68.2 % of participants were TAFE trainees rather than established workers, although the majority worked as apprentices on a part-time basis. Lastly, the survey was not conducted during the hottest part of the year, posing the opportunity for recall bias.

## Conclusion

This study provides important insights and baseline information regarding workers’ perceptions and attitudes towards workplace heat exposure. Workers in South Australia were moderately concerned about heat exposure. Further heat educational programmes and training should focus on those undertaking physically demanding work outdoors, in particular young workers and those over 55 years with low education level. The high proportion of respondents unsatisfied with current heat prevention measures and supporting more heat-related regulations indicates the necessity to refine current workplace heat prevention policies in a warming climate. In addition, there is a need to develop local specific and clear enforceable heat regulations.

## Abbreviations

CFMEU, Construction, Forestry, Mining and Energy Union; IPCC, intergovernmental panel on climate change; OH&S, occupational health and safety; OSHA, occupational safety and health administration; PPE, personal protective equipment; SWSA, SafeWork South Australia; TAFE, technical and further education

## Additional files

Additional file 1: Survey questionnaire: extreme heat exposure and worker health & safety. (PDF 256 kb) Additional file 2: Information sheet for participants. (DOC 130 kb)
