# Supplementary material for: Workers’ perceptions of climate change related extreme heat exposure in South Australia: a cross-sectional survey
Source: BMC Public Health. 2016 Jul 11;16:549. doi: 10.1186/s12889-016-3241-4 (PMC4940878; doi:10.1186/s12889-016-3241-4)

THE UNIVERSITY OF ADELAIDE AUSTRALIA

Questionnaire Number: ____________

Discipline of Public Health
School of Population Health & Clinical Practice

Extreme Heat Exposure and Worker Health & Safety

A South Australian Survey

General Instructions

- This survey, which is being conducted by The University of Adelaide, is investigating workplace heat exposure and occupational health & safety.
- Please answer each question as best you can by placing a (✓) in the box next to your preferred answer.
- For each question, please select one response unless otherwise instructed.
- Please follow any arrows or instructions that direct you to the next question.
- This survey is anonymous to maintain confidentiality.
- A reply paid envelope is enclosed for the return of completed questionnaire.

Thank you very much for taking the time to complete this questionnaire

Contact person: Jianjun Xiang; Email: jianjun.xiang@adelaide.edu.au
Phone: 8313 6875

1. **What is your gender?**

Male ☐ 1

Female ☐ 2

2. **What is your age?** ___________ (years)

3. **What is your level of formal education?**

High school ……………………….…. ☐ 1

Trade certificate…………………….. ☐ 2

University degree …....................... ☐ 3

Other (specify)…... ………………… ☐ 4

____

4. **What is your current main occupation?**

Technician and/or trades worker……….. ☐ 1

Health and/or community services worker ☐ 2

Clerical & administrative worker………… ☐ 3

Retail/Sales worker……………………… ☐ 4

Machinery operator and/or driver……….... ☐ 5

Labourer…………………………………… ☐ 6

Other (specify)______________________ ☐ 7

5. **What is the industry of your current employment?**

Agriculture, forestry & fishing……… ☐ 1

Mining……………………………….. ☐ 2

Manufacturing……………………….. ☐ 3

Construction…………………………. ☐ 4

Wholesale Trade & Retail Trade….. ☐ 5

Transport & Storage……………….. ☐ 6

Other (specify)__________________ ☐ 7

6. **In which type of workplace environment do you currently work?**

Completely indoors………… ☐ 1

Mainly indoors……………… ☐ 2

Completely outdoors………. ☐ 3

Mainly outdoors…………….. ☐ 4

Other (specify below)……… ☐ 5

____

7. **Would you consider your job to be physically demanding (e.g. lifting or moving heavy or awkward objects)**

(Please tick one box)

Not at all	*A little*	*Moderately*	*Very much*
1	*2*	*3*	*4*
☐	☐	☐	☐

8. **Do you work around heat sources like ovens, furnaces, or welding etc?**

Yes ☐ 1 →How often?

- Seldom……… ☐ 1
- Sometimes…. ☐ 2
- Often……..... ☐ 3
- Always……… ☐ 4

No ☐ 2

9. **Does your job require the use of protective clothing such as overalls?**

Yes ☐ 1 →Types? ______________________

→How often?

- Seldom…… ☐ 1
- Sometimes… ☐ 2
- Often……..... ☐ 3
- Always…… ☐ 4

No ☐ 2

10. Which of the following applies when you are working during very hot weather?

- I drink plenty of fluids before starting work ☐ 1
- I drink fluids regularly while at work……... ☐ 2
- I only drink when thirsty…………………… ☐ 3
- All of the above…………………………….. ☐ 4
- Other, please specify below……………… ☐ 5

____

11. What have been your main sources of information about preventing heat illnesses in the workplace? *(You can choose more than one)*

- Training………………. ☐ 1
- Internet……………….. ☐ 2
- TV and Radio……….... ☐ 3
- Newspaper…………..... ☐ 4
- Friends and families…. ☐ 5
- Colleagues………….... ☐ 6
- Workplace……………. ☐ 7
- SafeWork SA……….. ☐ 8
- None………………….. ☐ 9
- Others (specify below) ☐ 10

____

12. Have you ever experienced a heat illness (such as heat rashes, heat exhaustion or heat stroke etc) at work?

- Yes……... ☐ 1 →Types?
- No………. ☐ 2
- Not sure… ☐ 3

Types:
- Heat rashes……… ☐
- Heat cramps…….. ☐
- Heat exhaustion… ☐
- Heat stroke……… ☐
- Other……………. ☐

13. Have you been given instructions on first aid procedures for serious heat illnesses?

- Yes………. ☐ 1
- No………. . ☐ 2
- Not sure… ☐ 3

14. How concerned are you about the risk of heat illness at work during very hot weather?

(Please tick one box)

Not at all 1	A little 2	Moderately 3	Very much 4
☐	☐	☐	☐

15. Have you ever injured yourself at work during very hot weather?

- Yes……. ☐ 1 →If yes, please tick the type of injury?
- No…… ☐ 2
- Not sure ☐ 3

Type of injury:
- Falls, trips and slips………. ☐
- Hitting objects…………….. ☐
- Being hit by moving objects ☐
- Burn……………………….. ☐
- Other injuries……………… ☐

16. Have you witnessed an injury to another person during very hot weather?

- Yes……. ☐ 1 → If yes, please tick the type of injury?
- No…….. ☐ 2
- Not sure ☐ 3

Type of injury:
- Falls, trips and slips………. ☐
- Hitting objects…………….. ☐
- Being hit by moving objects ☐
- Burn……………………….. ☐
- Other injuries……………… ☐

17. Have you attended a training course which covers the prevention of heat illnesses?

Yes…………... ☐1
No……………. ☐2
Not sure…….. ☐3

18. Do you think there is a need for more training about working in very hot weather?

Yes………….. ☐1
No…………… ☐2
Don't know…. ☐3

19. Are there any guidelines in your workplace for working during very hot weather?

Yes…………. ☐1
No…………… ☐2
Don't know…. ☐3

20. Do you think there should be more legal requirements or restrictions for working during very hot weather?

Yes ☐1
No ☐2 →Because? (You can choose more than one)

- ► There are enough regulations…… ☐1
- ► I don't think it is a serious problem ☐2
- ► I haven't thought about it………… ☐3
- ► Other(specify)________________ ☐4

____

Don't know ☐3

21. What measures are adopted in your workplace during very hot weather?

(You can choose more than one)

- ► Provision of cool drinking water…………….. ☐1
- ► Electric fan…………………………………….. ☐2
- ► Broad brimmed hats supplied………………… ☐3
- ► Shady rest area………………………………… ☐4
- ►Stopping work if the temperature exceeds 40℃ ☐5
- ► Central cooling system or air conditioning…… ☐6
- ► Rescheduling work time, e.g., start work early, extend break time, etc…………………... ☐7
- ► Others (specify)________________________ ☐8

____

____

____

____

22. Are you satisfied or dissatisfied with the measures currently adopted in your workplace for reducing the risk of heat illnesses in very hot weather? (Please tick one box)

Strongly dissatisfied	*Dissatisfied*	*Undecided*	*Satisfied*	*Strongly satisfied*
1	*2*	*3*	*4*	*5*
☐	☐	☐	☐	☐

23. Do you generally work at your own pace during very hot weather?

Yes ☐ 1

No ☐ 2 → Because? (*<u>You can choose more than one</u>*)

- ► Enough has been done to cool the workplace ☐ 1
- ► Pressure from co-workers………… ☐ 2
- ► Pressure from supervisors………... ☐ 3
- ► Pressure from work demand……… ☐ 4
- ► Other (specify)________________ ☐ 5

____

24. Do you think you should adjust your work habits personally to reduce the risk of heat illnesses when working during very hot weather?

Yes ☐ 1

No ☐ 2 → Because? (*<u>You can choose more than one</u>*)

- ► I don't think it is a serious problem ☐ 1
- ► I don't think I am at risk…………… ☐ 2
- ► Enough has been done already….. ☐ 3
- ► I haven't thought about it………….. ☐ 4
- ► Other (specify)________________ ☐ 5

____

Don't know ☐ 3

25. If you have any comments you would like to make about workplace heat exposure and Occupational Health & Safety, please write them below.

____

____

____

____

____

THE END

Thank you for participation. Your contribution to this survey is very greatly appreciated.

Please return your questionnaire in the reply paid envelope provided.

If the envelope has been mislaid, please forward to:

Jianjun Xiang

Discipline of Public Health, The University of Adelaide, Level 8, Hughes Building, Mail Drop DX 650 207, Adelaide, SA 5005

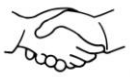

Supplement: Additional file 1: — Survey questionnaire: extreme heat exposure and worker health & safety. (PDF 256 kb) [file 12889_2016_3241_MOESM1_ESM.pdf]
