# Supplementary material for: Workers’ perceptions of climate change related extreme heat exposure in South Australia: a cross-sectional survey
Source: BMC Public Health. 2016 Jul 11;16:549. doi: 10.1186/s12889-016-3241-4 (PMC4940878; doi:10.1186/s12889-016-3241-4)
Supplement: Additional file 2: — Information sheet for participants. (DOC 130 kb) [file 12889_2016_3241_MOESM2_ESM.doc]

# **
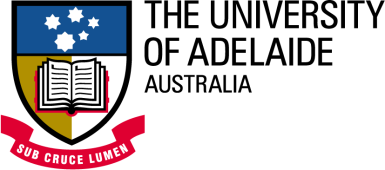
**

# DISCIPLINE OF PUBLIC HEALTH

Level 8, Hughes Building

North Terrace Campus

# **INFORMATION SHEET**

# **Extreme Heat Exposure and Workers’ Health & Safety in South Australia**

# Research Background


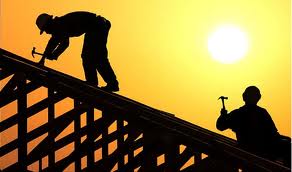

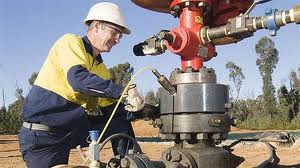
Occupational exposure to extreme heat may lead to adverse health effects and contribute to work-related injury. Those at risk of heat stress include outdoor workers and workers in hot environments such as those working outdoors in the building and construction trades, firefighters, agricultural workers, miners, boiler room workers and factory workers. With the predicted increase in the frequency and intensity of extremely hot weather in South Australia, workplace heat exposure is presenting a growing challenge to occupational health and safety.

# Aims of the research

This project aims to: (a) explore the extent to which manual workers are affected by or adapt to extreme heat exposure in South Australia; (b) identify which industrial sectors and occupations are most vulnerable to heat-related illness and injury in SA; (c) investigate how stakeholders and manual workers perceive the impact of workplace extreme heat exposure on OH&S and productivity loss; and (d) utilize the findings to inform policy implications and guidelines.

# Study Design

Using both qualitative and quantitative methods, this research will be conducted in Adelaide in three stages:

- Stage I is the analysis of historical OH&S data to explore the association of heat exposure and occupational compensation claims in SA.
- Stage II will ascertain views and experiences of various stakeholders and key informants regarding extreme heat in the workplace.
- Stage III is a questionnaire survey of workers to explore perceptions of workplace heat exposure, behavioural responses and adaptation barriers in South Australian workplaces.

# How do I participate?

If you are interested in this project and would like to participate, please email: [jianjun.xiang@aedelaide.edu.au](mailto:jianjun.xiang@aedelaide.edu.au). We are currently seeking workers to complete a questionnaire. Target workers are those mainly undertaking manual work outdoors or in hot environments. For this we require the permission and support of their employers. Questionnaires could be provided to employers for distribution to workers. Stamped and addressed envelopes will be provided for the return of completed questionnaire. Alternatively we could visit workplaces and conduct face-to-face surveys which would take about 5-10 minutes to complete. Participation is voluntary and people can withdraw from the study at any time without any reasons given.

# Confidentiality would be assured

The questionnaire is anonymous and neither worker nor workplace will be identifiable in the final analysis. Only members of the research team will have access to data. The study findings will be published in a completely unattributable format in order to ensure that no participants will be identified.

# What if I have a complaint or any concerns?

If you wish to raise concerns about the conduct of the project with an independent person or discuss matters related to the University policy on research involving human participants or your rights as a participant, contact: The Human Research Ethics Committee’s Secretary, Ph: 08 8303 6028 or visit: [http://www.adelaide.edu.au/ethics/human/guidelines/applications/#complaint](http://www.adelaide.edu.au/ethics/human/guidelines/applications/#complaint).

# For further information, please contact:

Jianjun Xiang, Discipline of Public Health, the University of Adelaide,

[jianjun.xiang@adelaide.edu.au](mailto:jianjun.xiang@adelaide.edu.au)

**Your contribution to this survey is very greatly appreciated.**

**
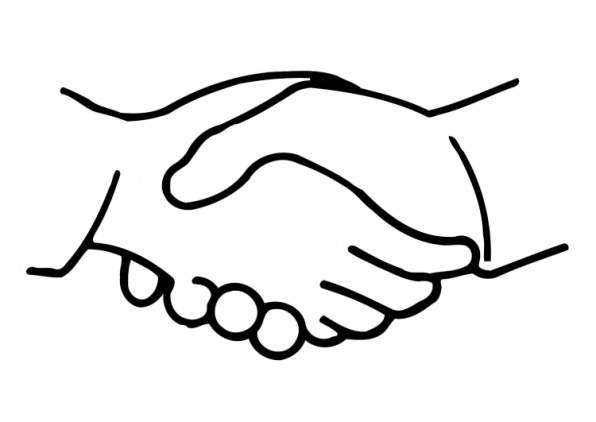
**
